# Aortic arch stiffness in Fabry disease

**DOI:** 10.1186/1532-429X-15-S1-E124

**Published:** 2013-01-30

**Authors:** Zoubir M Bensalah, Cédric Collin, Alban Redheuil, Pierre Boutouyrie, Dominique Germain, Elie Mousseaux

**Affiliations:** 1 Radiology, HOSPITAL AMBROISE PARE, Boulogne-Billancourt, France; 2 Cardiovascular Radiology, Hospital George Pompidou, Paris, France; 3 Departement of Pharmacology, Hospital George Pompidou, Paris, France; 4 Genetic, Hospital Raymond Poincaré, Garches, France

## Background

Background: Aortic thoracic remodelling has been recently described in FD, however no data was available concerning AAS in this rare genetic disease.

Aim of this study was to assess aortic arch stiffness (AAS) parameters in male patients with Fabry disease (FD) using cardiovascular magnetic resonance imaging (CMR).

## Methods

Twenty nine males with FD matched with 58 controls for age underwent CMR using cine and phase contrast velocity sequences.

Thoracic aortic diameter; local (distensibility, β-index stiffness), global (pulse wave velocity) stiffness parameters of the aortic arch and cardiac proprieties were assessed by CMR.

## Results

Aortic arch PWV was significantly increased in FD patients (6.5±3.1vs 5.0 ±1.5 m/s, p<0.01)

Compared to control subjects, patients with FD had also markedly decreased distensibility (2.73±1.14 vs 3.45±1.13 10-2 kPa-1, p<0.01) and increased stiffness index beta (9.4 ±6.7.10-2 vs 5.9±2.7.10-2, p<0.001) in the ascending aorta.

Descending aortic stiffness parameters were also impaired with a trend for decreased distensibility (2.26±1.15 vs 3.15±1.0 10-2 kPa-1, P=0.06) and significant increased for β-index stiffness (8.5 ±3.9 .10-2 vs 2.9±0.9.10-2, p<0.0001).

## Conclusions

FD patients exhibited impairment of both local and global aortic arch stiffness parameters.

## Funding

Nothing

